# A Novel Toll-Like Receptor 2 Agonist Protects Mice in a Prophylactic Treatment Model Against Challenge With *Bacillus anthracis*

**DOI:** 10.3389/fmicb.2022.803041

**Published:** 2022-03-14

**Authors:** Chih-Yuan Chiang, Douglas J. Lane, Yefen Zou, Tim Hoffman, Jianfeng Pan, Janice Hampton, Jillian Maginnis, Bishnu P. Nayak, Ugo D’Oro, Nicholas Valiante, Andrew T. Miller, Michael Cooke, Tom Wu, Sina Bavari, Rekha G. Panchal

**Affiliations:** ^1^Division of Molecular Biology, United States Army Medical Research Institute of Infectious Diseases, Frederick, MD, United States; ^2^Genomics Institute of the Novartis Research Foundation, San Diego, CA, United States; ^3^Novartis Vaccines and Diagnostics, Siena, Italy; ^4^Novartis Vaccines and Diagnostics, Cambridge, MA, United States

**Keywords:** TLR2, agonist, *Bacillus anthracis*, *in vivo*, prophylactic

## Abstract

Current therapies for anthrax include the use of antibiotics (i.e., doxycycline, and ciprofloxacin), an anthrax vaccine (BioThrax) and *Bacillus anthracis*-specific, monoclonal antibody (mAb) (i.e., Raxibacumab and obiltoxaximab). In this study, we investigated the activity of immunomodulators, which potentiate inflammatory responses through innate immune receptors. The rationale for the use of innate immune receptor agonists as adjunctive immunomodulators for infectious diseases is based on the concept that augmentation of host defense should promote the antimicrobial mechanism of the host. Our aim was to explore the anti-*B. anthracis* effector function of Toll-like receptor (TLR) agonists using a mouse model. Amongst the six TLR ligands tested, Pam_3_CSK_4_ (TLR1/2 ligand) was the best at protecting mice from lethal challenge of *B. anthracis.* We then evaluated the activity of a novel TLR2 ligand, DA-98-WW07. DA-98-WW07 demonstrated enhanced protection in *B. anthracis* infected mice. The surviving mice that received DA-98-WW07 when re-challenged with *B. anthracis* 20 days post the first infection showed increased survival rate. Moreover, ciprofloxacin, when treated in adjunct with a suboptimal concentration of DA-98-WW07 demonstrated augmented activity in protecting mice from *B. anthracis* infection. Taken together, we report the prophylactic treatment potential of DA-98-WW07 for anthrax and the utility of immunomodulators in combination with an antibiotic to treat infections caused by the *B. anthracis* bacterium.

## Introduction

*Bacillus anthracis*, the etiological agent of anthrax, is rod-shaped, gram-positive, and non-motile bacterium. *B. anthracis* exists in either vegetative or spore forms. The vegetative form of *B. anthracis* rarely causes diseases. The spore form of *B. anthracis* is infectious and highly resistant to inactivation. The spores can remain dormant in the environment for decades and infect grazing animals that ingest the spores. *B. anthracis* spores can be introduced into the host through ingestion, skin lesion and inhalation. Humans can acquire the disease following exposure to spores released purposely as a bioterrorist weapon or accidentally from naturally occurring sources. Given that inhalational anthrax is particularly deadly, *B. anthracis* is ranked near the top of the list for potential bio-threat agents.

After entering the host, *B. anthracis* spores germinate locally or at regional lymph nodes after being transported by phagocytic cells. *B. anthracis* upregulates toxin expression within hours of germination. Protective antigen (PA) and lethal factor (LF) combine to form lethal toxin (LT) whereas PA and edema factor (EF) combine to form edema toxin (ET) (Lovchik et al., 2012). Binding of the host cell surface receptor with the PA portion of the complex facilitates the translocation of toxins to the cytosol, in which EF and LF exert their toxic effects (Guidi-Rontani et al., 2000). LF, a zinc-dependent bacterial protease, cleaves mitogen-activated protein kinase kinases 1 and 2 (MEK1 and MEK2) and inactivates MAPK signal transduction pathway (Klimpel et al., 1994). EF, a highly active adenylyl cyclase, de-regulates cellular gene transcriptions through elevating cyclic AMP concentration within the intracellular compartment (Lorenz et al., 2000). The physiological impact of both ET and LT is the alteration of the peripheral vasculature. ET causes venous and arterial relaxation and LT can produce myocardial dysfunction and interfere with endothelial integrity (Suffredini et al., 2017).

Current standard of care for anthrax is a 60-day course of antibiotic (ciprofloxacin or doxycycline) treatment. The use of single or a combination of antibiotics will be determined based on the route of infection, the age and overall health of the patients and other factors (Mayo Clinic, 2017). Aggressive treatment such as mechanical ventilation and continuous fluid drainage may be accompanied. As the disease progresses, more toxin is produced in the host and hence overcomes the effect of the drugs. Two anthrax antitoxin drugs (Raxibacumab and obiltoxaximab) have been approved by the United States Food and Drug Administration (FDA) and are included in the Strategic National Stockpile for treating inhalational anthrax (Yamamoto et al., 2016). Both Raxibacumab and obiltoxaximab are mAbs that bind to PA and block its binding to anthrax toxin receptors and prevent the internalization of anthrax toxins (Froude et al., 2011). BioThrax, the only licensed anthrax vaccine, is indicated for pre-exposure prophylaxis of disease in persons at high risk of exposure and post-exposure prophylaxis of disease following suspected or confirmed *B. anthracis* exposure (Longstreth et al., 2016).

Recognition of microbial pathogens is an essential element for the activation of mammalian innate immune response. This is mediated by germline-encoded pattern recognition receptors (PRRs) that recognize pathogen-associated molecular patterns (PAMPs), molecular structures that are broadly shared by pathogens. Ligation of TLRs to their cognate ligands leads to pro-inflammatory and/or interferon responses. For example, TLR2 mediates the sensing of vegetative *B. anthracis* whereas nucleic acid sensing TLRs (TLR7 and TLR13 in mice and their human counterparts) detect high amounts of RNA present in the spore surface layer (Choo et al., 2017). The role of TLRs in bacterial infection was further elucidated in TLR deficient mice. TLR2-deficient mice had an increased bacterial burden and succumbed to *Staphylococcus aureus* infection (Takeuchi et al., 2000). Similarly, TLR2-deficient mice infected with *Listeria monocytogenes* showed reduced survival rate, increased bacterial burden and micro abscesses in the liver (Torres et al., 2004). The ability to activate anti-microbial activity through the TLR pathways suggests TLR-ligands could be utilized as an approach to fight against infectious disease. A combination of TLR2/6 (Pam_2_CSK_4_) and TLR9 (ODN2395) ligands resulted in survival of 100% of mice from lethal challenge of *Pseudomonas aeruginosa* (Duggan et al., 2011). The administration of the TLR2 ligand (MALP-2) prior to *Streptococcus pneumoniae* infection reduced pulmonary bacterial burden in mice (Reppe et al., 2009). Furthermore, intravitreal injection of Pam3Cys, a TLR2 ligand, protected mice from *S. aureus* endophthalmitis, preserved retinal structural integrity, and maintained visual function (Kumar et al., 2010).

In this study, we investigated the efficacy of a novel TLR2 ligand, DA-98-WW07, in protecting mice from lethal challenge of *B. anthracis*. Administration of a benchmark TLR1/TLR2 ligand, Pam_3_CSK_4_, prior to lethal *B. anthracis* infection also showed protective activity. Similar to the phenotype observed with Pam_3_CSK_4_, DA-98-WW07 also protected mice from *B. anthracis* infection. Notably, DA-98-WW07 was able to elicit prolonged protective activity. This was demonstrated in the experiment where infected mice that survived following DA-98-WW07 treatment, when re-challenged with *B. anthracis* 20 days post initial infection also showed increased survival. Moreover, adjunct therapy with the combination of ciprofloxacin and suboptimal concentration of DA-98-WW07 showed augmented activity in protecting mice from *B. anthracis* infection.

## Materials and Methods

### Mice

C57BL/6 wild type mice were purchased from Charles River, NCI colony, Frederick, MD. All animal studies were performed in the BSL-3 containment facilities at the United States Army Medical Research Institute of Infectious Diseases (USAMRIID).

### Ethics

Animal research at USAMRIID was conducted under an animal use protocol approved by the USAMRIID Institutional Animal Care and Use Committee (IACUC) in compliance with the Animal Welfare Act, PHS Policy, and other Federal statutes and regulations relating to animals and experiments involving animals. The facility is accredited by the Association for Assessment and Accreditation of Laboratory Animal Care International (AAALAC) and adheres to principles stated in the Guide for the Care and Use of Laboratory Animals (National Research Council, 2011).

### Reagents

Toll-like receptor (TLR) ligands Pam_2_CSK_4_ (tlrl-pm2s-1), Pam_3_CSK_4_ (tlrl-pms), Poly (I:C), lipopolysaccharide (LPS) (tlrl-pb5lps), R-848 (tlrl-r848) and ODN 1826 (tlrl-1826) were all purchased from Invivogen (San Diego, CA, United States). Ciprofloxacin was purchased from Sigma-Aldrich (St. Louis, MO, United States). DA-98-WW07 was synthesized at the Genomics Institute of the Novartis Research Foundation (GNF).

### Preparation of *Bacillus anthracis* Spores

Ames strain (pXO1^+^, pXO2^+^) of *B. anthracis* was prepared as previously described (Ivins et al., 1990). Approximately 10^3^ vegetative colony formation unit (CFU) was inoculated into 100 ml of Leighton-Doi broth. The culture was incubated for 18 h with vigorous shaking at 37°C. Two-liters Erlenmeyer flasks, each containing 200 ml of Leighton-Doi broth, were inoculated with 5-ml amounts of the culture. After the cultures were shaken for 24 h at 37°C, 800 ml of sterile distilled water was added to each flask, and incubation with shaking was continued for 40 h. The cells and spores were pelleted at 10,000 × *g* and then suspended in phosphate-buffered saline (PBS) containing 0.1% gelatin (PBSG). The cell suspension (10 ml) was added to 35-ml centrifuge tubes containing 8 ml of 60% Renografin (E. R. Squibb & Sons, Princeton, NJ, United States) overlaid with 14 ml of 50% Renografin in PBSG. These discontinuous gradients were centrifuged in a Sorvall HB-4 hanging bucket rotor (Du Pont Co., Wilmington, DE, United States) for 3 h at 12,000 × *g*. The spores, which formed a band at the interface of the 50 and 60% Renografin layers, were removed with a pipette and diluted with an equal volume of PBSG. The spores were pelleted at 10,000 × *g*, suspended in PBSG containing 12% glycerol, and frozen at −70°C.

### Production of Human Toll-Like Receptor 2 Expressing Nuclear Factor Kappa B-Luciferase Cell Line (HEK293-hTLR2-FLAG-Nuclear Factor Kappa B-Luc)

HEK293-Nuclear Factor Kappa B (NF-κB)-Luc cells (clone LP58), a cell line stably transfected with a reporter vector in which the luciferase gene is under the control of an NF-κB dependent promoter. These cells were transfected using lipofectamine 2000 (Thermo Fisher Scientific, Waltham, MA, United States) with hTLR2-FLAG plasmid encoding for human TLR2 containing a FLAG epitope at the C- terminus and a blasticidin resistance gene for selection according to manufacturer’s protocol. Transfected cells were cultured in the presence of selection antibiotics and individual resistant clones were picked, expanded, and tested for expression of luciferase upon stimulation with the TLR1/2 agonist Pam_3_CSK_4_, TLR4 agonist LPS, TLR7/8 agonist R848, TLR 2/6 agonist Pam_2_CSK_4_.

### Cell Culture

All cultured cells were grown at 37°C in a humidified environment containing 5% CO_2_. HEK293-hTLR2-FLAG-NF-κB-Luc cells were cultured in complete medium supplemented with puromycin (5 μg/ml) and blasticidin (10 μg/ml). Mouse cells from lymph nodes (LNs) and spleens were cultured in RPMI 1640 (Thermo Fisher Scientific, Waltham, MA, United States) supplemented with 1% Penicillin-Streptomycin, non-nssential Amino Acids, L-glutamine, Sodium Pyruvate and 10% heat-inactivated fetal calf serum (Hyclone).

### Human Peripheral Blood Mononuclear Cells Isolation

Human PBMCs were isolated from buffy coats of healthy donors using Ficoll gradient (Amersham, United Kingom) and cultured in RPMI 1640 (Thermo Fisher Scientific, Waltham, MA, United States) supplemented with 1% Penicillin-Streptomycin, non-nssential Amino Acids, L-glutamine, Sodium Pyruvate, HEPES and 10% heat-inactivated fetal bovine serum (Hyclone). Buffy coats from healthy donors were obtained from the Scripps Normal Blood donor program in La Jolla, California. Informed consent was obtained before all blood donations.

### *In vitro* Activity on Human Toll-Like Receptor 2 Stable Cells

For the luciferase reporter gene assay, HEK293-hTLR2-FLAG-NF-κB-Luc cells (25 × 10^3^ cells/well) were seeded into 384-well flat bottom plates. After 18 h incubation, cells were stimulated in duplicates with different concentrations of stimuli for 6 h. Subsequently, cells were lysed with 30 μl of Bright Glo Luciferase buffer (Promega, Madison, WI, United States) and luciferase levels were measured using the LMax II384 microplate reader (Molecular Devices, San Jose, CA, United States). Relative luminescence units (RLU) from each sample (average of 2) were divided by the RLU of the control sample (PBS or DMSO) and expressed as fold induction (FI).

### Measurements of Cytokine Production

All cytokine measurements in the supernatants of cultured cells were performed using Mesoscale Assay (Meso Scale Discovery, Rockville, MD, United States) to detect human or mouse cytokines following manufacturer’s instructions.

### Statistical Analyses

Kaplan-Meier survival curves were generated using Prism GraphPad software (Version 7.04) Survival rates were compared by Fisher exact test and time to death (days) were compared by Log-rank test.

### Experimental Design of the Efficacy Studies

**FIGURE 1:**
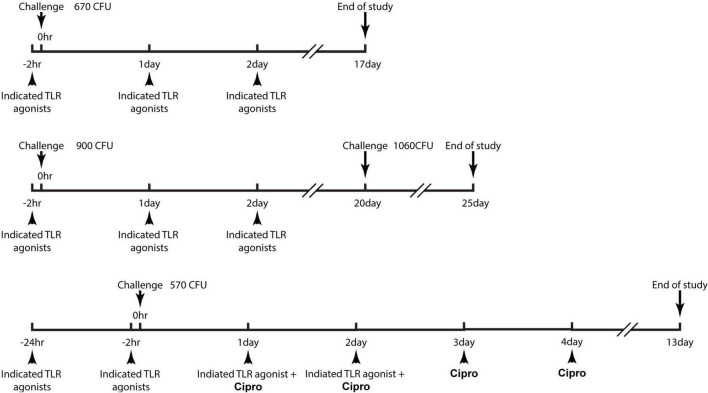
Schematic diagrams demonstrating the dose regimen of the studies. Detailed description of the study design can be referred to the “experimental design of the efficacy studies” in the materials and methods section.

**FIGURE 2:**
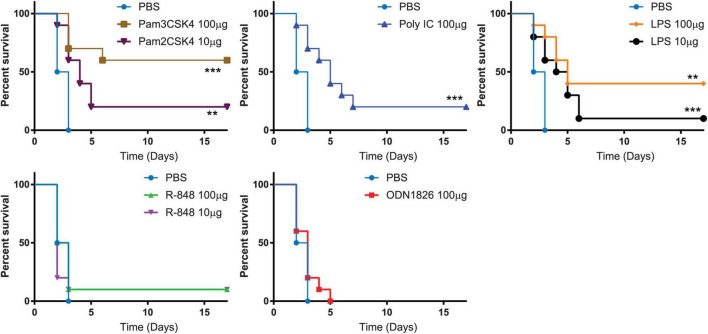
Toll-like receptor (TLR) agonists induced various level of resistance to *B. anthracis* infection. Kaplan-Meier curves of C57BL/6 mice infected with *B. anthracis*. Mice are pre-treated with indicated benchmark TLR agonists followed with *B. anthracis* infection. Statistically significant differences (Log-rank test) in time to death (days) between the treated verses the control are indicated. *** indicates *p*-value < 0.001. ** indicates *p*-value < 0.05.

The study designs, group designations, dosing regimens are provided in detail as Figure 1 and Table 1. C57BL/6 mice (∼20 gm) were infected with a target dose ranging from 670 to 900 CFU of *B. anthracis* (Ames strain) *via* the intraperitoneal route. In study 1 (Figure 2), TLR agonists were administered *via* intramuscular (i.m.) route to animals 2 h prior to infection, 24 and 48 h post infection. The survival of the mice was monitored for a course of 17 days. In study 2 (Figure 4), TLR agonists were administered *via* i.m. route to animals 2 h prior to infection, 24 and 48 h post infection. On day 20, surviving mice were re-challenged with *B. anthracis*. The survival of the mice was monitored for an additional 5 days. In study 3 (Figure 5) TLR agonists were administered *via* intraperitoneal (i.p) route to animals 24 and 2 h prior to infection, 24 and 48 h post infection. Ciprofloxacin was administered *via* the i.p route at 24, 48, 72, and 96 h post infection. The survival of the mice was monitored for a course of 13 days.

**TABLE 1:** Study designs.

Rodent study	Treatment route	Route of pathogen	Pathogen tested	CFU	Number of mice	Groups	Regimen
1	IM	IP	*B. anthracis* (Ames strain)	670	10 per group	1. PBS control	TLR agonists treatment (-2, 24, and 48 h)
						2. Pam_2_CSK_4_ (10 μg)	
						3. Pam_3_CSK_4_ (100 μg)	
						4. Poly IC (100 μg)	
						5. LPS (10 μg)	
						6. LPS (100 μg)	
						7. R-848 (10 μg)	
						8. R-848 (100 μg)	
						9. ODN1826 (100 μg)	
2	IM	IP	*B. anthracis* (Ames strain)	900	10 per group	1. PBS control	DA-98-WW07 treatments (-2, 24, and 48 h)
						2. DA-98-WW07 (10 μg)	
						3. DA-98-WW07 (30 μg)	
						4. DA-98-WW07 (100 μg)	
						5. DA-98-WW07 (300 μg)	
						6. Pam_3_CSK_4_ (300 μg)	
3	IP	IP	*B. anthracis* (Ames strain)	570	10 per group	1. PBS control 2. Ciprofloxacin (15 mg/kg) 3. DA-98-WW07 (5 μg) 4. DA-98-WW07 (5 μg) + Cipro (15 mg/kg)	Ciprofloxacin treatments (+24, +48, +72, and +96 h) DA-98-WW07 treatments (-24, -2, 24, and 48 h)

*IM, intramuscular; IP, intraperitoneal.*

**FIGURE 3:**
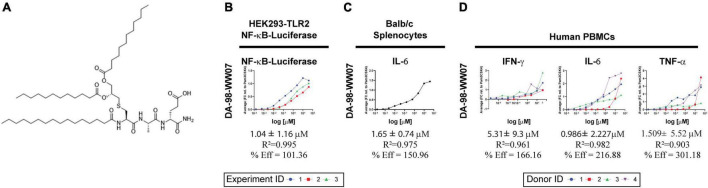
Activity of a novel TLR1/2 agonist, DA-98-WW07. **(A)** Structure of DA-98-WW07 (IUPAC Name) (4*R*,7*S*,10*R*,14*R*)-4-carbamoyl-14-(dodecanoyloxy)- 7-methyl-6,9,17-trioxo-10-palmitamido-16-oxa-12-thia-5,8-diazaoctacosanoic acid). **(B)** Luciferase expression in HEK293-cells stable transfected with FLAG-tagged human TLR2 and a NF-κB-luciferase reporter gene after stimulation with various doses of DA-98-WW07. Pam_3_CSK_4_ was used as a benchmark for the calculation of percent efficacy. The curves represent three independent experiments. The top curve was generated from only one data set, while the bottom curves were the best fit from two replicate data sets. **(C)** Murine splenocytes were stimulated with different concentration of DA-98-WW07. Secretion of IL-6 in the supernatants was measured as an indicator of DA-98-WW07 activity. The best fit curve from a single experiment is shown panel **(D)** Human PBMCs were stimulated with different concentrations of DA-98-WW07. Secretion of IFN-γ, TNF-α and IL-6 in the supernatants was measured as an indicator of DA-98-WW07 activity. The curves represent different donors.

**FIGURE 4:**
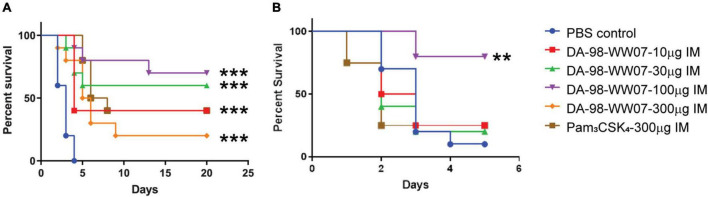
DA-98-WW07 protected mice from *B. anthracis* infection. Mice survival following infection with *B. anthracis*. Kaplan-Meier curves of C57BL/6 mice infected with *B. anthracis*. **(A)** Mice are treated with indicated TLR agonists 2 h prior to infection and 24 or 48 h post infection. **(B)** Surviving mice were re-challenged with *B. anthracis* 20 day post initial infection. The number of mice that were re-challenged in the different groups are DA-98-WW07 –10 μg (4 mice), DA-98-WW07 –30 μg (5 mice), DA-98-WW07 –100 μg (5 mice), Pam_3_CSK_4_- 300 μg (5 mice), and PBS control (10 mice). Statistically significant differences (Log-rank test) in time to death (days) between treated vs. control groups are indicated. ^***^ indicates *p*-value < 0.001. ^**^ indicates *p*-value < 0.05.

**FIGURE 5:**
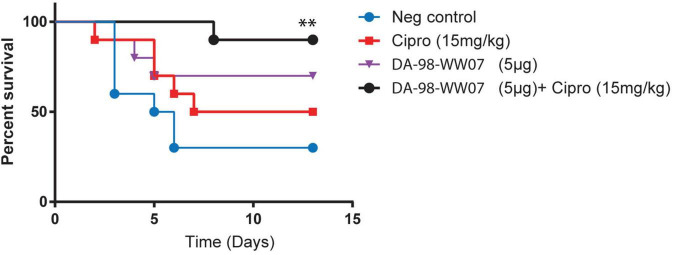
Ciprofloxacin, when treated in adjunct with sub-optimal doses of DA-98-WW07, augmented protective activity against *B. anthracis* infection. Kaplan-Meier curves of C57BL/6 infected with *B. anthracis*. Mice were treated with indicated TLR agonists 2 and 24 h prior to infection and 24 or 48 h post infection. Concomitantly, mice were not treated or treated with ciprofloxacin 24, 48, 72, and 96 h post infection. Statistically significant differences (Log- rank test) in time to death (days) between treated vs. control group are indicated. ^**^ indicates *p*-value < 0.05.

## Results

### Benchmark Toll-Like Receptor Agonists Induced Various Level of Resistance to *Bacillus anthracis* Infection

Synthetic TLR agonists were evaluated for their activities in protecting mice that received a lethal challenge dose of *B. anthracis*. Murine TLRs were stimulated with the following synthetic ligands: Pam_3_CSK_4_ (TLR1/2 agonist), Pam_2_CSK_4_ (TLR2/6 agonist), poly (I:C) (TLR3 agonist), LPS (TLR4 agonist), R848 (TLR7/8 agonist) or ODN1826 (TLR9 agonist). Each of the synthetic TLR agonists that were used in the study had reported concentration at which maximal cytokine secretion is stimulated from dendritic cells (Aliprantis et al., 1999; Krug et al., 2001; Lee et al., 2003; Yamamoto et al., 2003; Buwitt-Beckmann et al., 2005). Mice that received PBS diluent succumbed to *B. anthracis* infection 3 days post challenge. Mice that were treated with 100 μg Pam_3_CSK_4_ demonstrated a statistically significant (*p* = 0.0108; Fisher exact test) increase (60%) in survival rate and mean time to death (*p* = 0.0004; Log-rank test) compared to vehicle treated controls (0%), after challenge with *B. anthracis*. Mice treated with other TLR agonists showed the following survival rate: 10 μg of Pam_2_CSK_4_ (20%), 100 μg Poly (I:C) (20%), 10 μg of LPS (10%), 100 μg of LPS (40%), 10 μg of R848 (10%), 100 μg of R848 (10%), and 100 μg ODN1826 (0%) (Figure 2). The results suggest that potentiating the host immune response specifically by agonizing TLR2 protected mice from lethal infection of *B. anthracis*.

### DA-98-WW07 Is a Novel Agonist of Toll-Like Receptor 2

Previously, we have conducted a series of high-throughput screens on a chemical library of 1.8 million compounds intending to identify novel TLR2 agonists (Santone et al., 2015). This resulted in the identification of a group of triacetylated lipopeptides active on both human and mouse TLR2 which differed in the amino acid component and in the length of the acyl chain. This class of lipopeptide bears a triacylated cysteine glycerol core, similar to the known TLR2 agonist Pam_3_CSK_4_, but differs in the serine and lysine amino acid residues. A representative of this class of lipopeptides is DA-98-WW07 (Figure 3A). The dipeptide portion of DA-98-WW07 is composed of alanine and glutamic acid. Alanine can be substituted with alpha-aminobutyric acid with no apparent loss of activity (Santone et al., 2015). Glutamic acid, which is at the C-terminus of the lipopeptide, can tolerate even a wider range of chemical modifications. DA-98-WW07 induced NF-κB activity with EC_50_ of 1.04 ± 1.16 μM and its efficacy is 101.36% when benchmarked with Pam_3_CSK_4_, in the HEK293-hTLR2-FLAG-NF-κB-Luc reporter cell line (Figure 3B). TLR ligands such as LPS, Pam_2_CSK_4_, Pam_3_CSK_4_ exhibited a dose dependent induction of NF-kB activity when benchmarked with Pam_3_CSK_4_ in the luciferase reporter system (Supplementary Figure 1A). DA-98-WW07 also induced IL-6 secretion in murine BALB/c splenocytes with EC_50_ of 1.651 ± 0.744 μM and its efficacy is 150.96% when benchmarked with Pam_3_CSK_4_ (Figure 3C). Importantly, DA-98-WW07 up-regulated IFN-γ, TNF-α and IL-6 in human PBMCs with EC_50_ of 5.31 ± 9.3 μM, 1.509 ± 5.52 μM, and 0.986 ± 2.227 μM, respectively (Figure 3D). LPS, Pam_2_CSK_4_, Pam_3_CSK_4_ and R848 were able to induce aforementioned cytokine productions with varying potency (Supplementary Figure 1B). Together, these results suggest that DA-98-WW07 is a TLR2 agonist, active on both human and mouse receptors.

### DA-98-WW07 Demonstrated Protective Activity Against *Bacillus anthracis* Infection

The activity of DA-98-WW07 in protecting mice from lethal infection of *B. anthracis* was evaluated. Mice treated with the PBS vehicle succumbed to *B. anthracis* infection 4 days post challenge. Mice treated with 100 μg of DA-98-WW07 resulted in an optimal survival rate of 70%. However, mice treated with 10 μg, 30 μg and 300 μg of DA-98-WW07 resulted in 40, 60, and 20%, respectively. The activity of DA-98-WW07 was benchmarked with 300 μg of Pam_3_CSK_4_, which demonstrated 40% survival rate in this study (Figure 4A). To ascertain if mice cured from primary infection remained protected against subsequent *B. anthracis* inoculation, survivors from both DA-98-WW07 and Pam_3_CSK_4_-treated were re-challenged at day 20 with *B. anthracis* inoculum equivalent to the first infection and compared to a new untreated group of mice. Five days post re-challenge, mice (*N* = 5 mice) that received 100 μg of DA-98-WW07 in the first challenge study demonstrated 80% (4 out of 5 mice) of survival rate whereas mice that received Pam_3_CSK_4_ (*N* = 4 mice) had a 25% (1 out of 4 mice) survival rate (Figure 4B). Control mice (9/10 mice) succumbed to the infection by day 5. Altogether, this suggested DA-98-WW07 is able to elicit a protective effect when compared to Pam_3_CSK_4_.

### Ciprofloxacin, When Treated in Combination With a Sub-Optimal Dose of DA-98-WW07, Augmented Protective Activity Against *Bacillus anthracis* Infection

Treatment regimens incorporating therapeutics targeting both bacterial pathogens (i.e., the use of antibiotics) and host immune regulators is an emerging concept that has gained considerable research interest. Here, we investigated if the combination of DA-98-WW07 and ciprofloxacin, when both treated at suboptimal doses, would synergize the protective effect against a lethal infection of *B. anthracis*. Mice treated with 40 mg/kg of ciprofloxacin conferred 100% protection when challenged with *B. anthracis* (data not shown). When treated individually, 15 mg/kg of ciprofloxacin and 5 μg of DA-98-WW07 demonstrated 50 and 70% survival rate, respectively. Notably, the use of 15 mg/kg of ciprofloxacin as adjunct to 5 μg of DA-98-WW07 showed an increase to 90% survival rate (Figure 5). Although Pam_3_CSK_4_ was not evaluated in this study, the data suggests that TLR2 agonist in combination with an antibiotic could be a potential useful approach to augment survival in *B. anthracis* infected mice

## Discussion

Here, we report the use of TLR agonists, which potentiate the innate immune system, as an approach to combat *B. anthracis* infection. This investigation demonstrated TLR1/2 ligands, Pam_3_CSK_4_ and DA-98-WW07, provided beneficial prophylactic activity in protecting mice when challenged with *B. anthracis*. DA-98-WW07 protected mice from *B. anthracis* re-challenge 20 days after initial infection. Furthermore, Ciprofloxacin, when treated in combination with sub-optimal doses of DA-98-WW07, augmented protective activity against *B. anthracis* infection.

Due to the ease of generating antibiotic resistant strains of *B. anthracis* in the laboratory setting, the Secretary of the Department of Homeland Security issued a Material Threat Determination (MTD) specifically for multi-drug-resistant (MDR) anthrax (Athamna et al., 2004; Heine et al., 2017). Public Health Emergency Medical Countermeasures Enterprise (PHEMCE) also recognized MDR *B. anthracis* as a high-priority threat (Harris, 2013). Development of immunomodulators as an antibacterial therapy may provide an alternative to conventional antibiotics in the era of antimicrobial resistance (AR) (Institute of Medicine [IOM], 2012). Since immunomodulators directly target the host rather than the pathogen, they are unlikely to result in the development of antibiotic resistance even after repeated use. Furthermore, in situations where the etiological agent of disease is unknown, such as a bioterror attack, stimulation of innate immunity during the early stages of infection may be particularly useful as immune responses are often capable of providing protection against a broad range of pathogens. In particular, the threat of an intentional release of a highly virulent bacterial pathogen that is either intrinsically resistant to antibiotics, or has been weaponized *via* the introduction of antibiotic resistance, makes immunopotentiation an attractive complementary or alternative strategy to enhance resistance to bacterial biothreat agents.

Although immunomodulators have the potential to overcome AR and have a broad spectrum of antimicrobial activity, application of immunomodulators as an antibacterial treatment should be evaluated cautiously. First, protective pro-inflammatory, anti-microbial activity against pathogen-induced host tissue damage needs to be balanced. Although immunomodulators can result in the control of a pathogen, adverse events can sometimes accompany. Certain infections have been shown to trigger autoimmune disease and there is a possibility that the use of TLR agonists in susceptible individuals may promote the progression of autoimmunity. For example, CpG-ODNs have been implicated in triggering rheumatoid arthritis, systemic lupus erythematosus and diabetes (Duffy and O’Reilly, 2016). Patients who received PF4878691, a TLR7 agonist that was developed for treating hepatitis C virus, showed flu-like symptoms, lymphopenia and hypotension (Fidock et al., 2011). Assessing the benefit-risk ratio in situations of highly pathogenic infections which can rapidly deteriorate patients health condition should be taken into consideration. The adverse effect of DA-98-WW07 has not been evaluated in this study; however, we demonstrated the efficacy when combining immunomodulator at suboptimal concentration in adjunct with ciprofloxacin. Using immunomodulator at suboptimal concentration may circumvent the problem of over-activating the innate immune system and likely to reduce the incident of triggering autoimmune diseases. Second, several single-nucleotide polymorphisms (SNPs) have been identified in the coding regions of TLRs and their sensitivities to PAMPs or TLR agonist have been correlated. For example, arginine-to-glutamine substitution at residue 753 of TLR2 (TLR2 R753Q) showed diminished responsiveness to bacterial peptides derived from *Treponema pallidum* and *Borrelia burgdorferi* (Lorenz et al., 2000). TLR5^392*STOP*^, a polymorphism in the TLR5 ligand-binding domain, had defective responses to flagellin and increased susceptibility to *Legionella pneumophila* infection. Further study is required to understand the robustness of the host immune responses triggered by DA-98-WW07 in correlation with different TLR2 SNPs. Knowledge of the SNPs in any given individuals TLRs will be a pre-requisite in determining the use of any TLR agonist therapy (Medvedev, 2013). Third, some of the agents offer immediate protection which is independent of the route of inoculation while others are only effective when administered to the target organ such as the lung. One advantage of targeting organs such as lungs, is the possibility that treatments could be effectively self-administered, i.e., by intra-nasal administration. Determining the appropriate route of delivery is also important in advancing DA-98-WW07 for antibacterial therapy and intra-nasal delivery could potentially be beneficial in the case of inhaled anthrax.

BioThrax, the FDA approved anthrax vaccine, is manufactured from cell-free filtrates of cultures of a microaerophilic cultures of an avirulent, non-encapsulated strain of *Bacillus anthracis*. BioThrax primarily consists of PA that is adsorbed to aluminum hydroxide, which also serves as an adjuvant to enhance the immunogenicity of an antigen. Although BioThrax is effective, it requires prolonged dose regimen and compliance with the antimicrobial regimen has been problematic (Peachman et al., 2012; Hopkins et al., 2016). Combination of AV7909 [CPG 7909 (a synthetic immunostimulatory oligonucleotide)] with BioThrax boosted anthrax specific antibody responses of healthy subjects by 6–8 fold and accelerated the induction of immunity by approximately 3 week (Rynkiewicz et al., 2011). The potential use of DA-98-WW07 as a vaccine adjuvant in combination with BioThrax could also be investigated as an alternative therapeutic platform that requires further investigation.

While this study has mainly focused on the immunomodulatory role of TLR2 agonist in the prophylactic model of *B. anthracis* infection, a broader understanding of how TLR ligands modify bacterial and even viral infections is critical. Furthermore, additional experimentation will be needed to clearly map the role of various TLR ligands both *in vitro* and *in vivo* in the pre- and post-exposure infection models.

## Data Availability Statement

The raw data supporting the conclusions of this article will be made available by the authors, without undue reservation.

## Ethics Statement

The animal study was reviewed and approved by Animal Research at USAMRIID which was conducted under an animal use protocol approved by the USAMRIID Institutional Animal Care and Use Committee (IACUC) in compliance with the Animal Welfare Act, PHS Policy, and other Federal statutes and regulations relating to animals and experiments involving animals. The facility is accredited by the Association for Assessment and Accreditation of Laboratory Animal Care International (AAALAC), and adheres to principles stated in the Guide for the Care and Use of Laboratory Animals (National Research Council, 2011).

## Author Contributions

SB and RP conceived and coordinated the study. RP participated in the analysis and interpretation of the data. DL carried out the laboratory work. C-YC contributed to manuscript preparation. YZ, TW, JP, and TH identified and synthesized DA-98-WW07. AM, JM, JH, BN, and UD’O tested DA-98-WW07 in *in vitro* assays. NV, AM, and MC were TLR platform leaders at Novartis. All authors read and approved the final manuscript.

## Author Disclaimer

Opinions, interpretations, conclusions and recommendations stated within the article are those of the authors and are not necessarily endorsed by the U.S. Army. UD’O and NV were employees of Novartis Vaccines at the time of the study; in March 2015 the Novartis non-influenza Vaccines business was acquired by the GSK group of companies. UD’O is now employee of the GSK group of companies.

## Conflict of Interest

UD’O reports ownership of GSK shares. The remaining authors declare that the research was conducted in the absence of any commercial or financial relationships that could be construed as a potential conflict of interest.

## Publisher’s Note

All claims expressed in this article are solely those of the authors and do not necessarily represent those of their affiliated organizations, or those of the publisher, the editors and the reviewers. Any product that may be evaluated in this article, or claim that may be made by its manufacturer, is not guaranteed or endorsed by the publisher.

## Supplementary Material

Supplementary Figure 1 Profiling the activities of Toll-like receptor (TLR) ligands. **(A)** Luciferase expression in HEK293-cells stable transfected with FLAG-tagged human TLR2 and a NF-κB-luciferase reporter gene after stimulation with various doses of LPS, Pam_2_CSK_4_, Pam_3_CSK_4_ and R848. Pam_3_CSK_4_ was used as a benchmark for the calculation of percent efficacy. The curves represent three independent experiments. **(B)** Human PBMCs were stimulated with various doses of LPS, Pam_2_CSK_4_, Pam_3_CSK_4_ and R848. Secretion of IFN-γ, TNF-α and IL-6 in the supernatants was measured as an indicator of ligands’ activity. The curves represent different donors.
