# Supplementary figures and images for: A Novel Toll-Like Receptor 2 Agonist Protects Mice in a Prophylactic Treatment Model Against Challenge With Bacillus anthracis
Source: Front Microbiol. 2022 Mar 14;13:803041. doi: 10.3389/fmicb.2022.803041 (PMC8965344; doi:10.3389/fmicb.2022.803041)

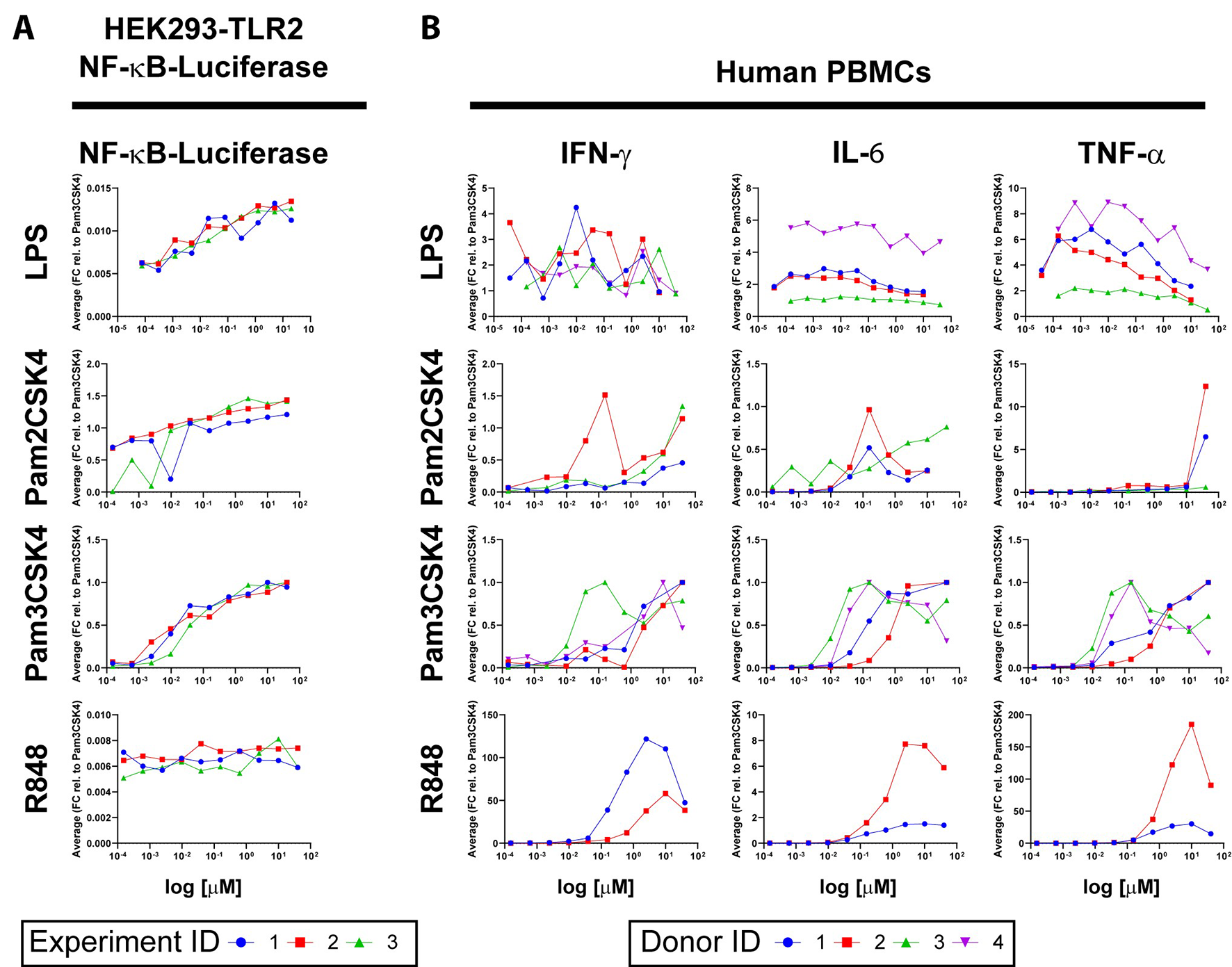

Supplement: Supplementary Figure 1 — Profiling the activities of Toll-like receptor (TLR) ligands. (A) Luciferase expression in HEK293-cells stable transfected with FLAG-tagged human TLR2 and a NF-κB-luciferase reporter gene after stimulation with various doses of LPS, Pam2CSK4, Pam3CSK4 and R848. Pam3CSK4 was used as a benchmark for the calculation of percent efficacy. The curves represent three independent experiments. (B) Human PBMCs were stimulated with various doses of LPS, Pam2CSK4, Pam3CSK4 and R848. Secretion of IFN-γ, TNF-α and IL-6 in the supernatants was measured as an indicator of ligands’ activity. The curves represent different donors. [file Image_1.TIF]
